# Sliding State Analysis of Fractal Rough Interface Based on the Finite Element Method

**DOI:** 10.3390/ma14092121

**Published:** 2021-04-22

**Authors:** Jianpeng Wu, Liyong Wang, Le Li, Yuechao Shu, Li Yang, Tonghui Lei

**Affiliations:** 1 The Key Laboratory of Modem Measurement and Control Technology in Ministry of Education, Beijing Information Science & Technology University, Beijing 100192, China; wangliyong@bistu.edu.cn (L.W.); shuyuechao@bistu.edu.cn (Y.S.); yangli@bistu.edu.cn (L.Y.); leitonghui@bistu.edu.cn (T.L.); 2 College of Mechanical and Electrical Engineering, Beijing Information Science & Technology University, Beijing 100192, China; lile@bistu.edu.cn

**Keywords:** friction pair, surface topography, finite element model, asperity

## Abstract

Local high temperature, stress concentration, and abnormal friction coefficients will appear at the friction pair in a wet clutch as a result of surface topography real-time changing. In order to improve the reliability of clutch friction components and reduce the failure phenomenon, the three-dimensional fractal surface topography data of the actual rough surface asperities are scanned, extracted, and processed successively, and then the finite element model of the rotary sliding friction pair is established considering the micro surface topography. Based on the finite element model, the variation of surface stress and strain is analyzed, and the friction coefficient measured experimentally is taken as the model input. It is concluded that when the rough surface and smooth surface make contact with each other, the maximum stress and plastic strain appear at the inner radius edge. Therefore, this research has a guiding significance for the structural design and processing technology of the friction components in a vehicle clutch.

## 1. Introduction

A wet friction pair is the main power transmission mode in a vehicle powertrain. Especially in the wet clutch, each friction pair is composed of a friction disc and steel disc. In their working process, the torque is transmitted by the sliding th friction between friction discs and steel discs [1,2,3]. However, the surface morphology of friction materials is random and complex. During the sliding friction process, the asperities make contact and deform with each other, and the resulting stress makes the asperities peel off and heat up [4,5,6]. Thus, the friction pair temperature field is increased, extending to pitting, buckling, wear, and so on [7,8]. However, it is difficult to observe the interface friction in these micro scales using equipment, which makes the interface morphology modeling and simulation of great significance [9,10,11]. On the other hand, exploring the influence mechanism of surface morphology on interface stress and friction characteristics can greatly reduce the friction pair failure and improve the material performance [12]. Our research is mainly aimed to improve the reliability of clutch friction components and to reduce the deformation and failure phenomenon.

Li [13] analyzed the radial and circumferential temperature field distribution on a friction disc surface by simulating the wet clutch sliding process, and the results showed that the radial temperature difference is the main cause of failure. Wang [14] investigated the distribution relationship between hot spot and contact pressure on one friction pair using the thermo-mechanical coupling finite element method, and the results showed that the temperature field is consistent with the contact pressure field. Wu [15] studied the influence of rotational speed and oil pressure on the temperature field during a stable period, and the conclusions provided guidance for the interface temperature field simulation. Khonsari [16] investigated the influence of different groove patterns (waffle shape, radial, and spiral) on the friction lining durability with a thermo-hydrodynamic analysis, and the results show that the friction material wear is directly related to the temperature and engagement load. Low metallic (LM), semi-metallic (SM), and non-asbestos organic (NAO) brake pads sliding against an iron disc were tested using a pin-on-disc tribometer [17]. From the fractal analysis, the fractal dimension of the brake pad surface was found to be in the range of 2.38–2.84 for all of the brake pads. Wu [18] studied the influence of circlip on non-uniform pressure distribution among the discs in the multi-disc clutches, pointing out that non-uniform pressure distribution is the main cause of deformation. Moreover, research on the phenomenon of uneven wear was also carried out by Han [19], which is based on the coupled thermo-mechanical analysis and shape optimization. Generally, research into this subject in recent years has mostly been based on the smooth interface temperature field or contact simulation with a single pair of asperities, which rarely reflect the overall surface topography characteristics and rarely combine with experiments. Therefore, the premise of accurate simulation of the friction interface should be characterizing the real surface morphology accurately, and obtaining the friction coefficient through experiments in order to ensure authenticity, which will be the main contribution of this paper.

In this paper, in order to systematically investigate the overall surface topography characteristics of the sliding friction pair, micro three-dimensional topography data are obtained through the digital scanning of the actual rough surface. After noise reduction, smoothing, and other mathematical processing, the mesoscopic finite element model of the sliding friction pair is approximately constructed considering the surface fractal characteristics. Then, the friction coefficient values of the wet friction pair are measured through the changing pressure experiment, which are used as a series of input parameters. Finally, the strain and stress are analyzed using a finite element model with different asperity heights and positions on the rough surface. The research results will have a guiding significance for the structural design and processing technology of the friction components in a vehicle clutch.

## 2. Feature Extraction

### 2.1. Fractal Theory

The research idea is to use the fractal principle for rough interface to zoom the complex rough surface in a relatively simple interface, so we first need to verify whether the rough surface has fractal characteristics. Majumdaret [20] used the Weierstrass–Mandelbrot (W–M) equation in fractal geometry to describe the surface microscopic profile:

(1) $$Z(x)=G^{\left(D-1\right)}\sum_{n=n1}^{\infty }\frac{\cos2\pi r^{n}x}{r^{(2-D)n}}(1<D<2,r>1)$$

In this formula, *D* is the contour fractal dimension; *G* is the characteristic length scale of surface microtopography, reflecting the magnitude of *Z*(*x*); *r^n^* is the lowest frequency, for rough surfaces, *r* = 1.5 and *r^n^* = 1/*L*; and *L* is the sampling length.

Then, we calculate the *D* and *G* by analyzing surface profile power spectrum, which can point out whether the surface contour is fractal. The power spectrum of the W–M equation can be approximated by the following continuous spectrum [21]:

(2) $$S(\omega )=\frac{G^{2(D-1)}}{2\ln r}\cdot \frac{1}{\omega^{\left(5-2D\right)}}$$

In this formula, *ω* is the spatial frequency of surface profile. Take the logarithm of both sides,

(3) $$\left\{\begin{array}{l} \mathrm{lg}S(\omega )=d+k\mathrm{lg}\omega \\ d=2(D-1)\mathrm{lg}G-\mathrm{lg}(2\ln r)\\ k=2D-5 \end{array}\right.$$

The two logarithmic functions of lg*S*(*ω*) and lg*ω* have a linear correspondence, and *k* is the gradient, and *d* is the intercept. Fractal parameters *D* and *G* are scale-independent, so they have obvious advantages in characterizing the fractal characteristics for rough surfaces [22,23].

If the power spectrum is a straight line and *k* satisfies −3 < *k* < -1, the contour is fractal [20]. Then, the fractal parameter *D* is calculated as follows:

(4) $$D=\frac{k_{p}+5}{2}$$

The fractal parameter g can also be calculated by Equation (3).

### 2.2. Surface Feature Extraction

Using the optical interferometer (Contour-GT), the surface topography of the wet clutch friction disc is measured. The Contour-GT is equipped with the data-display software Vision 64. The three-dimensional topographic data obtained by the optical interferometer are the coordinate values of all points on the friction disc surface, and the huge amount of data interferes with the rapid modeling and fractal feature verification. Therefore, it is necessary to extract part of all data for processing, and then fit a representative two-dimensional curve to facilitate the model construction.

**Figure 1:**
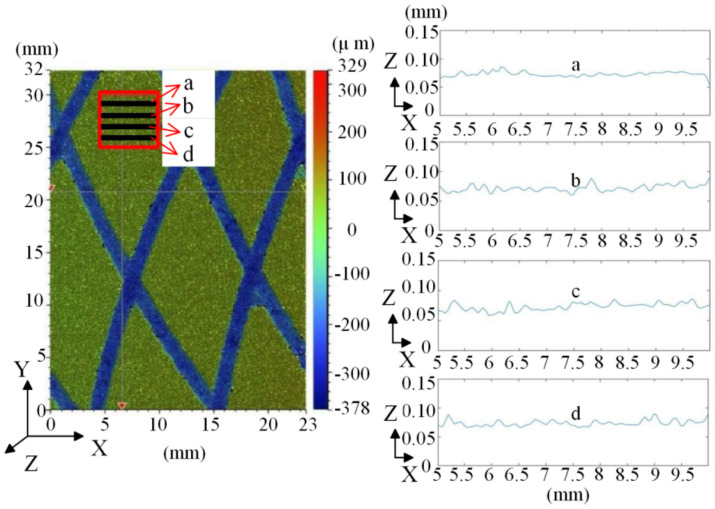
Surface topography and surface topographic curves.

A 5 mm × 5 mm area is randomly selected on the measured surface. As shown in Figure 1, four equidistant lines, a, b, c, and d, are selected on their vertical coordinates, and the topographic curves of these four lines are extracted to judge the fractal characteristics. In other words, the graph a–d presents the relative height of the microscopic surface.

At a micrometer scale, the fractal dimension of the friction disc is *D* = 1.34 after calculation, and the characteristic scale factor is *G* = e^−5.15^ (μm). When the fractal dimension is larger, *D* = 1.40, the characteristic scale factor is larger, *G* = e^−3.73^ (μm). In addition, the surface morphology is extracted seperately in four different friction stages, and the fractal dimension average values are 1.37, 1.36, 1.38, and 1.38, respectively, in the four stages, which are relatively stable. The scanning results prove that the friction disc has a fractal characteristic and the fractal characteristic is stable.

### 2.3. Data Processing

To facilitate the modeling, the five-spot triple smoothing method [24] is used to filter the real surface topology data, as shown in Figure 1.

The five-spot triple smoothing method can improve the authenticity and accuracy of the data [25], and the main procedure is selecting the five measured points, namely (*X*_t − 2_, *X*_t − 1_, *X*_t_, *X*_t + 1_, and *X*_t + 2_), and then fitting the data points by a polynomial. Suppose this polynomial is as follows:

(5) $$Q(t)=a_{0}+a_{1}+a_{2}t^{2}+\ldots +a_{n}t^{n}$$

(6) $$t=\frac{X_{t}-X_{t-1}}{h}$$

In this formula, *h* is the distance between two nodes. Through the calculation of the least square method, the value of *t* can be obtained as follows:

(7) $$\sum_{i=-n}^{n}R_{i}{}^{2}={\sum_{i=-n}^{n}\left[\sum_{j=0}^{m}a_{1}t_{i}{}^{j}-Y_{i}\right]}^{2}=\varphi (a_{0},a_{1},\ldots ,a_{m})$$

To make *ϕ* reach the minimum, deduce the following equation:

(8) $$\sum_{i=-n}^{n}Y_{j}t_{i}{}^{k1}=\sum_{j=0}^{m}a_{j}\sum_{i=-n}^{n}t_{i}{}^{k1+1}$$

In this formula, *k*1 = 0, 1, 2, ..., m. Because it is a five-spot method, take *n* = 2 and *m* = 3 to get all of the required values of *a*, and then put them into Equation (5) to get the fitting curve. As shown in Figure 1, it presents four 2D topographic curves after the least square fitting. Then, we can use the two-dimensional surface topography to reconstruct the surface topography, as the fractal features are universal on the rough surface.

## 3. Three-Dimensional Finite Element Model

**Figure 2:**
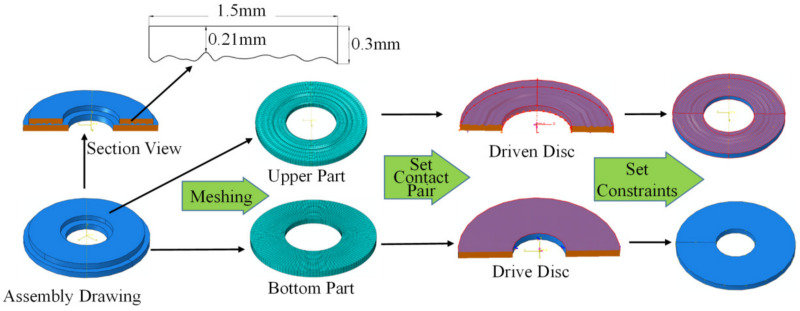
Modeling process of the friction pair.

In the three-dimensional model construction, the length is 1.5 mm, and the thickness is 0.21 mm–0.3 mm, which is reduced in equal proportion according to the actual vehicle wet clutch friction disc size. Figure 2 is the finite element modeling process diagram of the friction pair.

The 3D rough surface with grooves is generated by a 360-degree rotation of one 2-D topographic curve obtained by the fractal feature extraction above, and the distance between the rotational axis and the inner radius edge is 1.0 mm. We import the 3D rough surface model into the finite element analysis software ABAQUS as the upper part. The bottom part is simplified into a smooth disc. There is a clearance between the rough surface upper part and the bottom part when assembling. So, the two components constitute the whole ABAQUS friction pair model. The upper part has 16,640 elements, and the mesh type is C3D8R; the bottom part has 4914 elements, and the mesh type is the same as the upper part. The structural dimensions and material properties of the two parts are shown in Table 1, corresponding to the properties of the friction disc and steel disc in the wet clutch, respectively. As the bottom surface area is larger and its grid is coarser, the bottom part is selected as the drive disc for the friction pair. Meanwhile, the upper part is selected as the slave disc or driven disc. Therefore, the freedom degree is almost zero, except for the upper part of the *Y*-axis displacement, while the rotational angular velocity of the bottom part will be set.

The loading process of the finite element model is divided into two steps. The first step applies a uniform force to the upper part, so that the rough surface gradually approaches the bottom part; the second step applies a rotating load to the bottom part. When setting the freedom degree around the axis (*Y* axis) for the bottom part, the axis should be defined as the center of rotation. In the first step, all of the freedom degrees of the reference point are constrained. In the second step, the rotation angular velocity around the axis is set to 105 rad/s, and the other freedom degrees of the reference point remain at zero.

When the contact attribute is set, the penalty function should correspond to the field output in order to obtain an accurate convergence value. We will use the experimental method to measure the friction coefficient following pressure changes.

## 4. Test-Based Friction Coefficients

### 4.1. Equipment and Scheme

**Figure 3:**
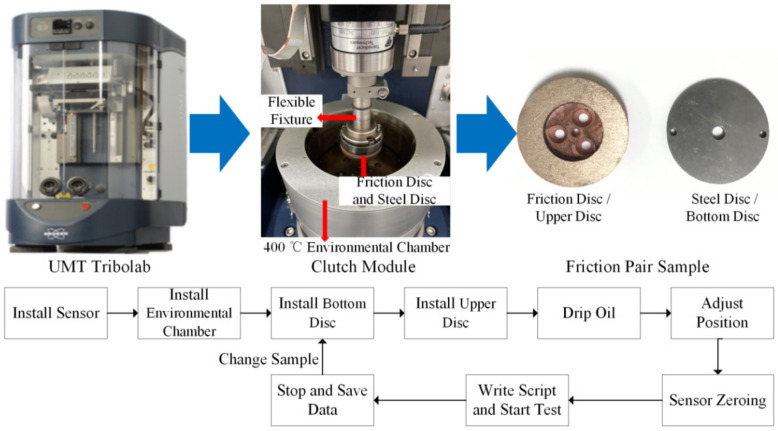
Sliding test.

In order to optimize the input parameters of the model, the UMT Tribolab clutch module is used to carry out a ring-disc friction test, and the friction coefficient is measured following the pressure changes. A universal mechanical tester (UMT), also called a tribometer, adopts the contact friction test form and is divided into upper and bottom modules. The upper module is responsible for the force application and the bottom module gives the motion mode. The force and speed data collected by the acquisition card are processed in real time by the computer software to obtain the data. Figure 3 is the test process diagram.

The UMT clutch module was selected for the test, including s 400 ℃ environmental chamber, 2000 N force sensor, and sample fixture. The physical structure parameters of the friction pair are shown in Table 2. The upper sample is a copper-based powder friction disc, while the bottom sample material is 65 Mn. The lubricating oil used in the experiment can be stored in the environmental chamber to provide a lubrication and cooling effect. The diameter of the environmental chamber is 70 mm, with a depth of 20 mm, and the amount of oil that can be stored in it is about 77 mL.

### 4.2. Equipmental Method

The lubricating oil should be dripped into the environmental cavity before the test, and we can carry out the sliding test according to the test steps, as shown in Figure 3. After installing the upper and bottom specimens, first adjust the position of the upper and bottom specimens to ensure alignment. Then, zero the sensor and write the run script. After the start of the sliding, the experimental data can be saved until the end of the experiment. Finally, the components are removed or replaced to start the next test.

### 4.3. Test Results

**Figure 4:**
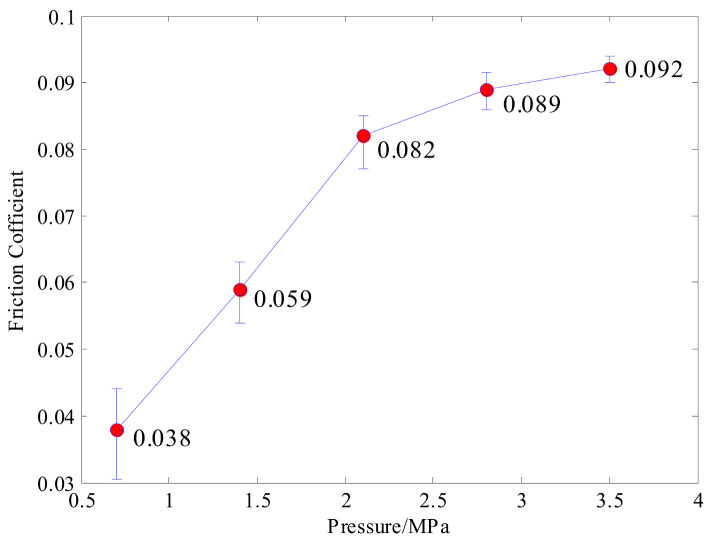
Friction coefficient versus pressure.

Figure 4 shows the friction coefficient changes following with different pressures which are measured in the experiment. We can see that the friction coefficient increases with raising pressure, which is between 0.038 and 0.092, but the growth rate decreases gradually. Moreover, the larger the pressure is, the smaller the friction coefficient error is. The values are taken into the penalty function in the contact property setting, to formulate the tangential behavior properties of the finite element model during contact.

## 5. Simulation and Analysis

**Figure 5:**
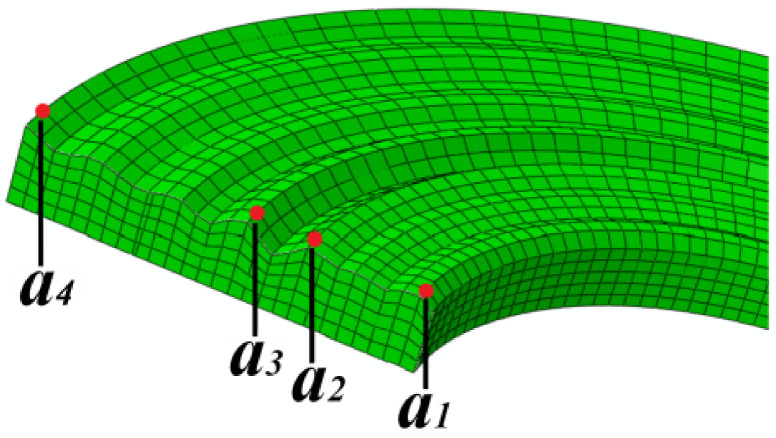
Reference node selection.

In order to analyze the simulation results in detail, four nodes are selected to extract the simulation results at different positions on the rough surface. The four peak nodes are selected as node *a*_1_, node *a*_2_, node *a*_3_, and node *a*_4_. The positions of each node are shown in Figure 5. It can be seen that node *a*_4_ is the highest peak of the upper part, and node *a*_1_ is the lowest asperity peak.

### 5.1. Stress Analysis

**Figure 6:**
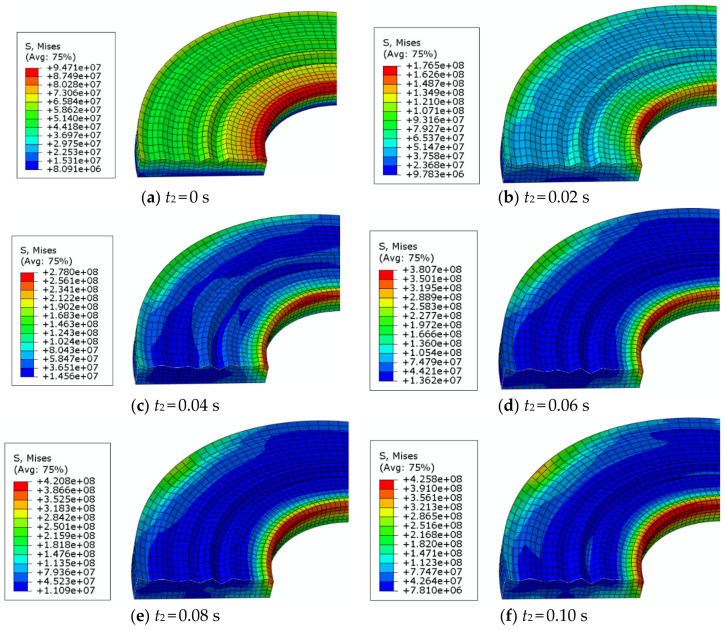
Stress of the upper rough surface.

In the frictional contact between the upper and bottom parts, the asperity will produce stress and strain because of the combined action of the pressure and speed. As shown in Figure 6, the total stress distribution on the upper surface gradually changes at each time point with 1.3 MPa pressure and 105 rad/s speed.

It can be seen from the Figure 6 that the stress concentration parts are generally distributed on the outer and inner edges of the rough surface, and the stress is mostly concentrated in the inner radius, where the asperity height is lower. This is because the surface height is lower on the edge, and the lower asperity will be squeezed or stretched by the higher asperity. Furthermore, the inner edge stress distribution is uniform along the circumference. The outer edge of the disc surface also has a stress concentration, but the distribution along the circumference is not uniform.

When the bottom part is stationary, all of the uniformly distributed forces on the upper part have been applied, as shown in Figure 6a. Under the force, the rough surface and the bottom part extrude each other, resulting in uniform stress distribution. At this time, the stress generated on the rough surface decreases with the increase in the radius, that is, the maximum stress is at the inner edge of the upper surface. The reason is that the radius there is the smallest, the asperities are squeezed by the higher asperities, and the stress is not easy to disperse. When the bottom part begins to rotate, as shown in Figure 6b–f, the uniformly distributed stress begins to appear, and the stress value increases with time.

**Figure 7:**
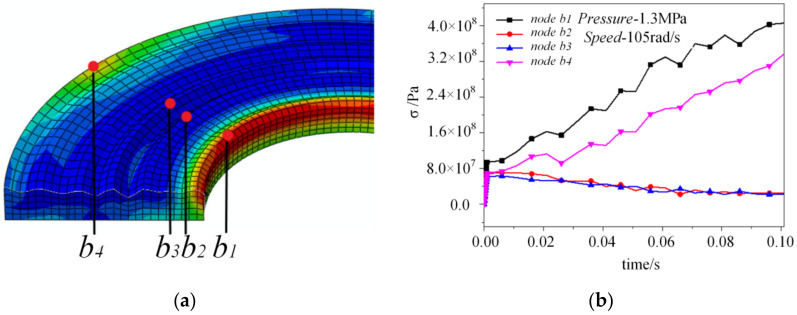
Stress analysis for the upper part. (**a**) Reference nodes for stress analysis. (**b**) Stress curve of nodes.

In order to analyze the stress concentration more clearly, the four nodes corresponding to the four asperities are extracted from the result stress nephogram, as shown in Figure 7a,b is the four nodes’ stress curve in the second analysis step with 1.3 MPa pressure and 105 rad/s speed.

As shown in Figure 7b, at the inner edge of the rough surface, that is, the node with the lowest height, the stress generated is always greater than that for the other three nodes. It can also be found from the figure. that the generated stress at the inner and outer edges will gradually increase with time, and the stress at the inner edge fluctuates greatly. However, the stress of the other two nodes is basically the same as for the middle radius, and has a downward trend with time. This is because the asperity in the middle radius can share the stress with its surroundings, so that the generated stress is dispersed. Furthermore, the stress difference between the outer edge and the inner edge keeps stable, which is about 70 MPa. However, the maximum stress difference between the middle radius and the outer edge can reach about 380 MPa. The inner edge asperity bears the extrusion or tension of the higher asperity along the radial direction after being loaded, so that the stress is larger and the distribution is uniform. This research result is very similar to other existing literature result figures [13], which are usually ignored in the research.

### 5.2. Strain Analysis

**Figure 8:**
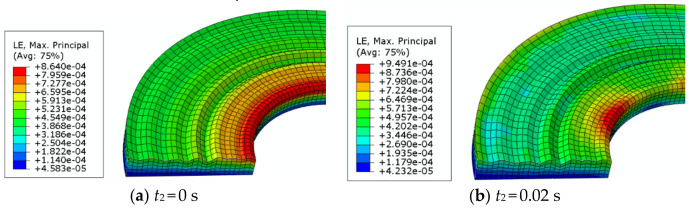

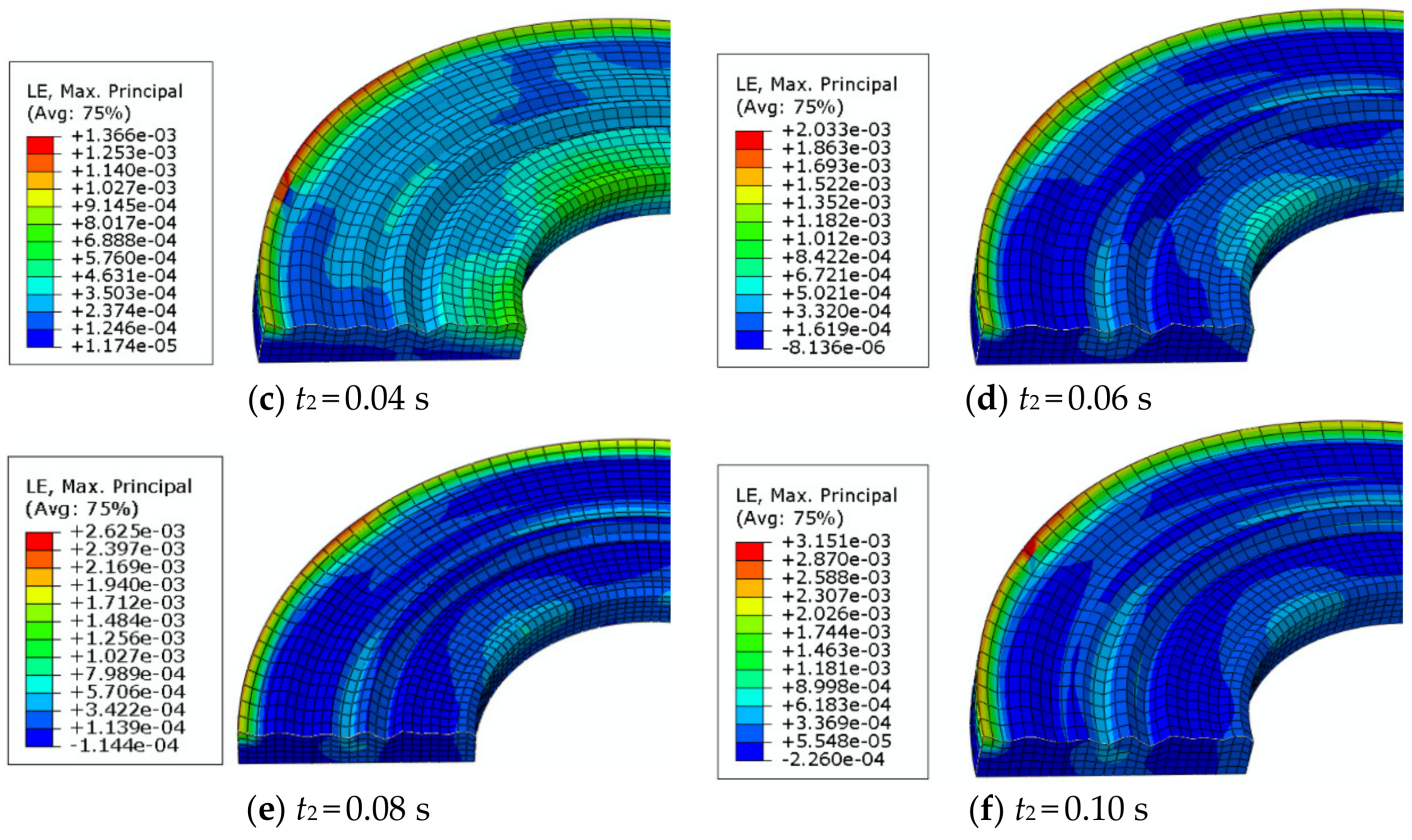
Strain analysis of the friction pair.

Figure 8 shows the strain nephogram in the second analysis step, which is generated during the sliding between the rough surface and the smooth surface. The pressure is 1.3 MPa and the speed is 105 rad/s. The contact is stable before the bottom part starts to rotate. At this time, the strain is small, and the maximum value appears at the dedendum of the inner edge asperity. The strain decreases with the increase in radius. As can be seen from Figure 8, the outer edge strain gradually increases with the sliding time, and the increasing rate is fast. When *t* = 0.04 s, the strain at the outer edge begins to be greater than that at the inner edge.

**Figure 9:**
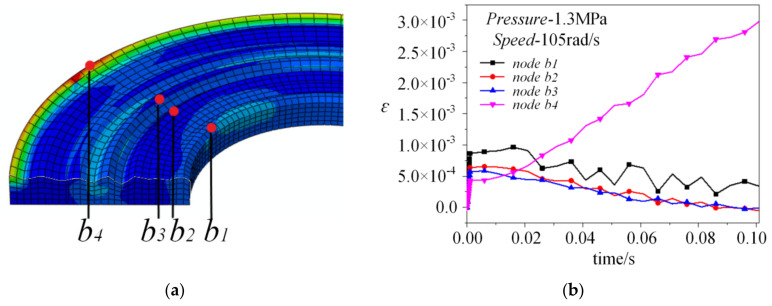
Strain analysis for the upper part. (**a**) Reference nodes for strain analysis. (**b**) Strain curve of the nodes.

Taking the same four nodes as the reference nodes for the strain analysis, the strain–time curves of four asperity peaks are obtained, as shown in Figure 9. It can be seen from Figure 7 that the stress at the outer edge and the inner edge increases gradually with time, and the stress generated at the inner edge is always higher. However, in Figure 9, only the outer edge strain increases with the sliding time, while the inner edge strain decreases, accompanied by large fluctuations. So, the outer edge strain is larger than the inner edge strain in the later stage. At the same time, in the middle radius region of rough surface, the strain always decreases steadily.

According to the strain formula:

(9) $$\varepsilon =\frac{L-L_{0}}{L_{0}}$$

In the formula, *L*_0_ is the original length and *L* is the length after deformation. At the inner edge, the original height of the asperity is lower. Under pressure, the asperity deformation at the inner radius is also small because of the small space. Therefore, there is little difference between the height after deformation and the original height. Thus, it is impossible to produce a large strain because of the increase in stress. The outer edge has a larger space to produce the corresponding strain. The original asperity height is quite different from that of the height after deformation. Therefore, the strain increases with the elevated stress at the outer edge.

### 5.3. Plastic Strain Analysis

**Figure 10:**
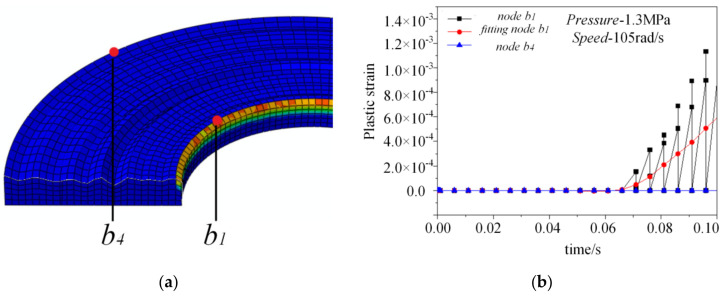
Plastic strain analysis for the upper part. (**a**) Reference nodes for plastic strain analysis. (**b**) Plastic strain curve of nodes.

Because the strengthening rate of the metal material is high and the strengthening rate changes very little, the linear hardening elastic-plastic model is selected. The corresponding relationship between the yield stress and plastic strain for the upper part is set as 500 MPa–0, 700 MPa–0.1, and 900 MPa–0.2. As shown in Figure 10a, the plastic strain of the upper part is obtained when the pressure is 1.3 MPa and the rotational speed is 105 rad/s. As only the inner edge appears to have a plastic strain, the plastic strain of the inner edge node *b*_1_ and the outer edge node *b*_4_ are extracted. The plastic strain curve of these two nodes is shown in Figure 10b. When the sliding time is 0–0.06 s, the plastic strain does not appear on the rough surface. When the sliding time reaches 0.065 s, a small area of plastic strain appears at the inner edge. Referring to Figure 7, it can be seen that the stress of the inner edge of node *b*_1_ is about 360 MPa, while the stress of the outer edge node is always less than 330 MPa. With the increase of inner edge stress, its plastic strain increases correspondingly.

Therefore, the plastic strain first occurs at the inner edge, and due to the increasing stress, the plastic strain increases with the sliding time. For the outer edge, the plastic strain is always zero in the whole sliding process, that is, there is no plastic deformation. Reviewing the above, the inner edge stress increases with time, while the strain decreases. The results show that the stress–strain relationship is no longer linear, so a plastic strain is produced, which is consistent with the plastic strain results in Figure 10.

In order to prove that this conclusion is not caused by the special surface profile, we compared the different surface profiles. Under the same working conditions (1.3 MPa pressure and 105 rad/s speed), the stress, strain, and plastic strain data of the *b*_1_, *b*_2_, *b*_3_, and *b*_4_ nodes at 0.1 s are obtained by sliding friction simulation, which are statistically analyzed in the table. The selection positions of the four nodes are consistent. It can be seen from Table 3 that the stress concentration is still at the inner edge and outer edge, the maximum stress difference between the middle radius and the outer edge can reach about 380 MPa. The strain at the outer edge is the largest, and inner edge takes is the second greatest. While the plastic strain at the inner edge is about 7 × 10^−4^, other areas remain zero at 0.1 s, which is really similar to the case above. Therefore, compared with the different surface samples, the experimental results are suitable for all of the microscopic surface topography analyses.

The conclusion of this paper is only limited to the specific component size and processing technology. To get a universal conclusion, we need to expand the number of samples, which is what we need to do in the future. Through the research results, we can see that the stress of the inner radius and outer radius is higher, and the plastic deformation problem of the inner radius is more serious. Therefore, in the engineering application, we can strengthen the metal at the inner and outer radius in order to obtain a longer service life.

## 6. Conclusions

In this paper, based on fractal theory, an optical interferometer is used to scan and extract the 3D topographic data on the rough surface. Then, we construct a finite element model of the sliding friction pair, considering the microscopic surface topography. The friction coefficient following the pressure is measured according to the experiment, and is taken into the penalty function of the contact attribute in the model. The stress and strain fields are obtained by simulation, and are then investigated for their changing rules during the working process of the wet friction pair. The conclusions are as follows:

1. At a microscopic scale, when the rough surface and the smooth surface of the two modeled discs are in contact with each other, the maximum stress is distributed on the inner edge, and its inner edge stress is about 70 MPa larger than the highest asperity at the outer edge. However, the strain generated at the inner edge will not be very large, because the deformation space is small. As it is stretched and compressed by all of the asperities in the radial direction, the inner edge stress continues to rise, and certain plastic strain will occur.
2. For the outer edge asperities, its deformation space is large, and its ability to disperse stress is also strong. Therefore, the stress of the outer edge asperity is not the maximum of the whole rough surface. Under the continuous action of external pressure, the strain increases linearly.
3. When the upper and bottom parts are in rotational sliding contact, the position where the plastic strain first occurs is not the highest asperity. Because of the shape, there will be largely concentrated stress in the inner radius with lower asperities. When the inner edge stress reaches about 360 MPa, plastic strain begins to appear. However, in the whole sliding simulation, the maximum stress of the outer edge is about 330 MPa, so there is no plastic strain at the outer edge. Compared with the different surface samples, the experimental results are suitable for all of the microscopic surface topography analyses.

## Figures and Tables

**Table 1:** Structure and material property parameters of finite element friction pair.

Parameter Setting	Upper Part	Bottom Part
Outer radius (mm)	2.75	3.0
Internal radius (mm)	1.25	1.0
Maximum thickness (mm)	0.3	0.3
Density ρ/(kg/m^3^)	5600	7800
Specific heat c/(J/(kg·°C))	536	487
Thermal conductivity λ/(W/(m·℃))	30	53
Thermal expansivity/(10^−5^/℃)	1.3	1.27
Elastic modulus E/(10^11^Pa)	1.1	1.6
Poissons ratio μ	0.30	0.30
Yield strengh σ_s_/MPa	500	356

**Table 2:** Physical structure parameters of the friction pair.

Friction Pair	Outer Radius/mm	Internal Radius/mm	Thickness/mm
Upper part	40.6	22	3.6
Bottom part	40.6	22	2.9

**Table 3:** Stress strain analysis for different profiles.

Profiles	Notes	Stress (10^8^ Pa)	Strain (10^−3^)	Plastic Strain (10^−4^)
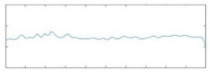	b_1_	3.94	0.38	6.87
	b_2_	0.19	<0.01	<0.01
	b_3_	0.22	<0.01	<0.01
	b_4_	3.18	2.51	<0.01
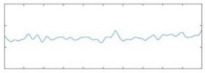	b_1_	4.02	0.41	7.21
	b_2_	0.27	<0.01	<0.01
	b_3_	0.20	<0.01	<0.01
	b_4_	3.26	2.74	<0.01
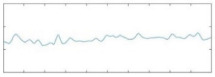	b_1_	4.11	0.42	6.98
	b_2_	0.29	<0.01	<0.01
	b_3_	0.21	<0.01	<0.01
	b_4_	3.45	2.63	<0.01
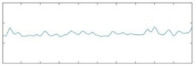	b_1_	3.89	0.37	6.45
	b_2_	0.18	<0.01	<0.01
	b_3_	0.25	<0.01	<0.01
	b_4_	3.23	2.56	<0.01

## Data Availability

The data presented in this study are available on request from the corresponding author.

## References

[1] Ingram M., Reddyhoff T., Spikes H.A. (2010). Thermal Behaviour of a Slipping Wet Clutch Contact. Tribol. Lett..

[2] Wu J., Ma B., Li H., Liu J. (2019). Creeping control strategy for Direct Shift Gearbox based on the investigation of temperature variation of the wet multi-plate clutch. Proc. Inst. Mech. Eng. Part D J. Automob. Eng..

[3] Wu J., Ma B., Li H., Wang L. (2021). The Temperature Field of Friction Disc in Wet Clutch Involving the Unconventional Heat Dissipation on the Contact Surface. Tribol. Trans..

[4] Cho J., Lee Y., Kim W., Jang S. (2018). Wet Single Clutch Engagement Behaviors in the Dual-Clutch Transmission System. Int. J. Automot. Technol..

[5] Wang Y., Li Y., Liu Y., Zhang W. (2019). Modeling and experimental research on engaging characteristics of wet clutch. Ind. Lubr. Tribol..

[6] Wang Y., Ren S., Li Y. (2018). Modeling of the drag torque of the disengaged grooved wet clutches with waviness. Proc. Inst. Mech. Eng. Part J J. Eng. Tribol..

[7] Katharina V., Hermann P., Karsten S. (2020). Running-in behavior of wet multi-plate clutches: Introduction of a new test method for investigation and characterization. Chin. J. Mech. Eng..

[8] Li T.-C., Huang Y.-W., Lin J.-F. (2016). Studies on centrifugal clutch judder behavior and the design of frictional lining materials. Mech. Syst. Signal Process..

[9] Deng T., Hu F.B., He Z.Y., Yin Y.L. (2019). Simulation, experimental testing and optimization of starting and shifting control Strategies of DCT wet dual clutches with respect to sliding friction. Iran. J. Sci. Technol. Trans. Mech. Eng..

[10] Yu L., Ma B., Chen M., Li H., Liu J., Zheng L. (2019). Numerical and experimental studies on the characteristics of friction torque based on wet paper-based clutches. Tribol. Int..

[11] Yang W., Tang X. (2017). Numerical analysis for heat transfer laws of a wet multi-disk clutch during transient contact. Int. J. Nonlinear Sci. Numer. Simul..

[12] Zhao E., Ma B., Li H. (2018). The tribological characteristics of cu-based friction pairs in a wet multidisk clutch under nonu-niform contact. J. Tribol..

[13] Li L., Li H., Wang L. (2017). Numerical analysis of dynamic characteristics of wet friction temperature fields. Adv. Mech. Eng..

[14] Wang L., Chen X., Li L. (2017). Research on wear calculation method and experiment for friction disc of wet clutch. J. Guangxi Univ. (Nat. Sci. Ed.).

[15] Wu J., Ma B., Li H., Liu J. (2019). Temperature rise characteristic of friction disc in wet clutch considering the local heat dissipation on the contact surface. J. Beijing Inst. Technol..

[16] Li M., Khonsari M., McCarthy D., Lundin J. (2017). Parametric analysis of wear factors of a wet clutch friction material with different groove patterns. Proc. Inst. Mech. Eng. Part J J. Eng. Tribol..

[17] Wei L., Choy Y.S., Cheung C.S. (2019). A study of brake contact pairs under different friction conditions with respect to charac-teristics of brake pad surfaces. Tribol. Int..

[18] Wu J., Ma B., Li H., Li M. (2018). The effect of circlip induced contact pressure on the temperature distribution in multi-disc clutch-es. Int. J. Veh. Des..

[19] Han M.J., Lee C.H., Park T.W., Son S.M. (2017). Coupled thermo-mechanical analysis and shape optimization for reducing uneven wear of brake pads. Int. J. Automot. Technol..

[20] Majumdar A., Bhushan B. (1990). Role of Fractal Geometry in Roughness Characterization and Contact Mechanics of Surfaces. J. Tribol..

[21] Berry M.V., Lewis Z.V. (1980). On the Weierstrass-Mandelbrot fractal function. Proc. R. Soc. Lond. Ser. A Math. Phys. Sci..

[22] Zuo X., Tang X., Zhou Y. (2020). Influence of sampling length on estimated fractal dimension of surface profile. Chaos Solitons Fractals.

[23] Liu Y., Wang Y., Chen X., Zhang C., Tan Y. (2017). Two-stage method for fractal dimension calculation of the mechanical equipment rough surface profile based on fractal theory. Chaos Solitons Fractals.

[24] Tang J., Fang J., Zhang Y. Study on the FOG’s signal based on wavelet. Proceedings of the International Symposium on Instrumentation & Control Technology: Signal Analysis.

[25] Zhi D., Jin S., Tianze L., Wang Y., Gao M. (2018). Filtering-Tikhonov regularization inversion for dynamic light scattering data with strong noise. Opt. Commun..

